# Abstracts and Gleanings

**Published:** 1879-07-20

**Authors:** 


### ABSTRACTS AND GLEANINGS.

**Effect of Posture in the Treatment of Strangulated and Incarcerated Hernia.**—*Case* 1. In August, 1834, I was called to see an infant male child having a strangulated, congenital, inguinal hernia, the strangulation having taken place the day previous. The child was much prostrated and was vomiting. After a prolonged and ineffectual attempt at reduction, I directed the mother, while the child was lying upon his back, upon a pillow, *with his feet and nates elevated*, to apply a bladder filled with cold water. No farther taxis was employed, and in about four hours the hernia retired.

*Case* 2. March 10, 1854, I was called lo see Samuel Tollhurst, æt. 2, having an indirect, inguinal, congenital hernia, which had been strangulated about twelve hours, during which time the mother had made ineffectual efforts to reduce it.

While the child was lying upon his back, upon a pillow, the mother by my direction, seized both feet and *raised the hips until nothing but the shoulders rested upon the pillow*. I continued to employ moderate taxis, and almost immediately the hernia disappeared.

*Case* 3. A male convict in Blackwell's Island Penitentiary, during the summer of 1875, while I was on duty at the Charity Hospital, had an indirect inguinal hernia which had become strangulated. When visited by me in the morning, the strangulation had existed several hours, and one of the house surgeons of Charity Hospital had been with him all night, making ineffectual attempts at reduction by taxis. The hernia was large and tender, and the condition of the patient was alarming. The patient himself wished to take an emetic, by which means, he said, it had once been reduced when strangulated. I did not think it wise to adopt his suggestion; but directed that while the house surgeon was making preparations for the operation, an attempt should be made to reduce the hernia by posture.

Accordingly *the foot of the bed was lifted, and its legs placed on the top of a dining table*, and, while the patient was lying supine, upon this very steep inclined plane, with his head down, moderate pressure was made upon the hernia. It began to diminish in size almost immediately, and in about ten minutes it disappeared altogether.

*Case* 4. Some years ago, and prior to the date of the case last recorded, a German, of middle age, living a few miles from Buffalo, N. Y., had a strangulated inguinal hernia. My former pupil, Dr. Ernest Pupikofer, was in charge, and had tried ordinary taxis for some hours before I arrived. I repeated the attempt and failed also. I then adopted the method described in case 3, and in a few minutes the hernia retired.

*Case* 5. August 2, 1873, I was requested to see Mr. ——, of this city, Drs. B. and F. in attendance. Three days before—July 31—he had been suddenly seized with pain in the region of the gall bladder which seemed to indicate the passage of a gall stone. Thirty-four hours before I saw him, while vomiting, a hernia descended through the inguinal ring. He never had a hernia before.

After prolonged taxis, the application of ice, and the employment of other judicious measures without success, Dr. W——, one of our most experienced surgeons, was added to the consultation, and the efforts at reduction were renewed. Subsequently, it becoming apparent that an operation could not be delayed much longer, Dr. B—— made the necessary preparations, and I was called.

At my suggestion Mr. —— *was laid upon a blanket upon the floor, and two men lifted his feet and legs upon their shoulders, while I made taxis.* This attempt failed. We then put him under the influence of ether, and a repetition of the same manœuvre was followed by almost immediate success.

*Case* 6. A German, aged about 40 years, had suffered from a reducible, indirect, inguinal hernia about ten years, which was sometimes reduced with difficulty. April 13, 1871, it came down and he was unable to reduce it, and on the following day he was sent to Bellevue Hospital. My house surgeon, Dr. Mitchell, gave him gr. ¼ of morphine, applied ice bags, employed taxis and raised the foot of the bed two feet.

*April* 15.—About forty-eight hours after the incarceration took place, he was not suffering from symptoms of strangulation; but the hernia which now occupied the scrotum, and appeared to be intestinal wholly, could not be reduced by taxis.

I then brought him before the class of medical students, put him under the influence of chloroform, elevated the hips upon pillows, and in about five minutes, under moderate taxis, the hernia retired.—*Dr. Hamilton, N. Y. Academy of Medicine.—Hospital Gazette.*

**Euonymus Atropurpurens.**—Dr. J. R. Black, in the Journal of Materia Medica, writes: As I have been in the habit of prescribing euonymus almost exclusively for lingering disorders of the digestive organs, and in the form of the fluid extract, it is of its therapeutical effects under such circumstances that I am able to write understandingly. Taken in appropriate doses, the immediate effects are slightly stimulant, almost like an exhilarant, which may last from one to three or four hours. Not, be it understood, similar to alcoholic stimulation, but a far milder and more durable feeling of vigor—not succeeded by the reaction of depression. The effect is far more like a quick acting tonic. In thinking of this, however, I am at a loss to discern whether this subordinate effect is really the outcome of an enhanced exaltation of vital action, or whether the more potent effects of a proper dose, in slightly morbid states, may not arise from its virtues in freeing the *primæ viæ* of the depression of inaction, or from the presence of effete matters inimical to a buoyant life. Be this as it may, the heavy, languid depression, the indisposition to mental or muscular action, often disappear like magic in certain morbid states of the digestive organs, under its influence.

Taken in doses of half a drachm to a drachm, according to individual susceptibility, before breakfast, the euonymus acts as a gastric tonic, cholagogue and mild cathartic, and usually within two hours after its administration. Its action is unattended by nausea, griping or any debilitating influence. This is its effect, not in the stereotyped, vague impression on some, but on those in whom there has been present a more or less lingering gastric, hepatic and intestinal torpor. Administered to subacute cases of functional inactivity of those parts, the dose has sometimes to be repeated every three or four hours before desirable action is re-established, when the effect is far more that of an active, frequently repeated cathartic. The stools indicate the large presence of bile, and of the feculent secretion of the intestinal follicles. It does not cause, like podophyllin, violent vermicular motions of the stomach and bowels, thereby producing nausea, griping and large discharges of gelatinous mucus, through irritant action. It is pre-eminently the most unobjectionable of cathartics to overcome habitually loaded abdominal viscera, not, however, in acute exacerbations, but in those of a more lingering character. Especially is it of eminent service when, from a variety of causes, hepatic and intestinal torpor are conjoined. In these instances it should not, of course, be given more than once daily, and in amount sufficient to produce not more than one or two evacuations. Continued use does not habituate and blunt the system to its energy, consequently the dose does not require to be increased; in fact, as a rule, rather decreased.

For habitual constipation, two rules should be observed in its administration: first, to give only enough to produce one or, at most, two evacuations per day; second, to order it to be taken with regularity at a certain time of each diurnal period. In the morning before breakfast is that usually preferred. The objects of these rules are to avoid begetting debility by over-action, and to establish a habit of regular feculation. Of course, special indications require special combinations, of which nux vomica, in certain states of motory enervation, is an example; but for ordinary and simple cases the following is a convenient form:

℞. Ext. euonymi, fluid............................f. ℥ iij.
Syr. aurantii..................................f. ℥ j. M.
Sig. One teaspoonful before breakfast.

If the debility and torpor are somewhat extreme, a dose may be taken before each meal, in which case half a teaspoonful is almost always amply sufficient.

From careful comparison, the euonymus has precisely the same therapeutic virtues as the so-called cascara sagrada, only decidedly more active, not only as a tonic, but as a corrector of hepatic languor and defective intestinal secretion. In large doses it is capable of hydragogue effect, perhaps less distressing and prostrating than almost any other agent producing a like effect. It is here proper to remark that the fluid extracts in the market are of unequal strength.

In summing up, it may be said that in euonymus we have an invaluable gastric tonic, mild cholagogue, pleasant laxative, or cathartic, according to the dose. It is admirably adapted for correcting deficient secretion and excretion of the intestinal follicles, for toning gastric atony of a non-inflammatory type, and for accelerating biliary outflow, and so ridding the system of the depressing effects of an almost habitual hydro-carbonaceous excess. It is not a diuretic, nor an alterative, any more than any remedy with similar effects is an alterative. It is not, as a rule, adapted to the treatment of acute, or even subacute diseases, but for many of the lingering forms it is simply invaluable.

**Opening the Mastoid Process by Surgical Procedure.**—In previous reports mention has been made of articles by Prof. Schwartze on this subject, which have been appearing in the Archiv. fur Ohrenheilkunde since 1872. They are now completed, and form a most valuable scientific investigation of the whole subject, based upon his observation of fifty cases.

He begins with a review of the history of the procedure which was known for a long time as Jasser's operation, from Dr. Jasser, a Prussian military surgeon, who performed it in 1776. In reality, however, it had been performed already by J. L. Petit, who died in 1750; he bored through the healthy bone and evacuated decomposed pus from the mastoid cells, and seems to have partially appreciated the value of the operation both on caries of the mastoid and on chronic otorrhea. Jasser, almost accidentally, opened a carious mastoid with a probe, and was greatly shocked to find that water syringed into the opening ran from the nose; but as the result of the procedure was very favorable upon the ear disease, he did the same operation upon the other ear, with the result of curing the chronic otorrhea which existed there. Great expectations were now formed that the operation would relieve all forms of deafness, but on account of disappointment in this respect it soon fell into disrepute, yet was tried occasionally as a last resource, till Von Bergen, a prominent Danish physician, who desired it performed on himself as a relief to deafness, dizziness and subjective noises, died from purulent miningitis, the result of perforating the brain instead of the mastoid cells.

In cases where sequestra exist in a mastoid without external symptoms, Schwartze considers the operation useful, but the difficulty is in making the diagnosis of this condition. Pain, fever, and a decidedly offensive odor to the otorrheal secretion, in spite of the most careful cleansing and disinfection of the tympanum and Eustachian tube, point to the existence of retained pus in the cells.

The operation as a prophylactic measure merely, to relieve chronic suppuration of the tympanum, and to avoid the possible dangers of pyemia, meningitis and tuberculosis, as suggested by Von Troeltsch and Jacoby, is considered by Schwartze of doubtful justification, on account of the risks of the operation and the possibility of anomalies in the formation of the parts involved. The operation is, however, an *indicatio vitalis* in these cases whenever symptoms of irritation of the brain are noticed.—DR. GREEN, *in Boston Medical and Surgical Jourual.*

**A Troublesome Parasite.**—Madgie P., three years of age, whose home is in a neighborhood where, in the spring and fall, almost every case of sickness needs anti-malarial treatment, had been suffering, for seven or eight days, during last October, with a quotidian intermittent. After the old women's remedies had been exhausted I was sent for, and, as a matter of course, diagnosed the case malarial fever. The symptoms were—fever every afternoon, lasting until morning and followed by profuse sweats; bowels alternately constipated and relaxed; tongue somewhat dry and furred; continued thirst; disgust for all food except milk; rapid emaciation; extreme debility.

Never during my experience has a case presented itself which called more loudly and plainly and persistently for specific treatment. With emphatic assurances to the family that on the morrow, or at the furthest, the next day but one, their child would be transformed from this miserable state into something like its former self, I ordered the following :

℞. Quinia sulphat............................grs. xvj.
Vini pepsini;
Syr. glycerr., comp..................aa. f. ℥j. M.
Sig. Teaspoonful every two hours, as directed.

Whether all physicians experience at times that "soft and subtle" consciousness—that "*Mens sibi conscia recti*"—steal upon them and suffuse their inmost selves, I do not know; but at the time of writing the above prescription I felt it, and pictured to myself how pleased the parents would be to see their only child so soon relieved. My visit was made between breakfast and dinner. The next day, toward evening, I again visited my patient, and came upon her in the act of taking a dose of medicine, which, almost as soon as swallowed, was ejected. This had been the case, I was informed, every time it was taken. She had a high fever, which came on at the usual time, and all the symptoms were "*in statu quo ante bellum.*" After giving orders to administer half the quantity of medicine, I left. Every day for two weeks my visits found the patient no better. Quinia, cinchonidia, cinchonia, followed by acid. hydrochlor. dil. and liq. potas. arsenit., with tinct. aconiti, liq. potas. cit., etc., during the fever, were given persistently, sometimes being retained and sometimes rejected by the stomach. Finally the child's parents were so emphatic in their denunciation of the "nasty medicine," that, having in mind a vision of homeopathy and its sweetness stepping into my shoes, I left some parvules of calomel, one-tenth grain each (prepared by Wm. R. Warner & Co.), to be given one every two hours. The next day I approached the house with fear and trembling, expecting to find my little patient sinking. What was my surprise on being greeted by the mother with, "Doctor, the baby is much better, and had no fever yesterday!" She at the same time handed me a tumbler containing a large *ascaris lumbricoides* (round worm), which the child had passed the day before, after taking three or four parvules. The nausea had also disappeared. After this I paid but three more visits, and the child speedily recovered.—DR. SHIVERS, *in Medical and Surgical Reporter.*

**Propylamine Chlorid.**—As there have been various opinions given latterly in regard to the efficacy of propylamine in the treatment of acute inflammatory rheumatism, I deem it not amiss to give my experience with it. I commenced the use of it some twenty years ago, soon after the reports of its use and success in the Pennsylvania Hospital, and for several years procured the drug from Bullock & Crenshaw, who manufactured it largely at that time. My first case was a young man aged nineteen, who had been suffering for three days intensely, and using colchicum without relief. I will not go into details and occupy your space by giving symptoms with which every intelligent practitioner is familiar, but simply say he had the disease in its worst form, every muscle and joint in his body seemed to be affected. He begged of me, when I entered the room, not to shake the floor or touch him. I prepared and gave the medicine in accordance with the formula used at the Pennsylvania Hospital, to-wit:

℞. Propylamine chlorid. ...............gr. 36.
Sach. alba..............................℥ij.
Aqua menth pip.........................℥vj. M. f. sol.

and directed him to take a tablespoonful every two hours. He commenced taking at at 10 a.m. I saw him next day at about the same hour; he was sitting in a chair, and, to use his own words, said, "What is the doctor coming here for? There is nobody sick here." He said he felt a little pain in one shoulder was all. I directed him to continue the medicine every four hours and stay in his room. In less than a week he went to ploughing and never had another attack. This is only one of upwards of fifty cases, many of them of the worst type, that have been entirely relieved in from four to fourteen days, very few of them over ten days, and in no case have I found the disease to return or leave any heart complication, unless the mischief had been done to that organ before the treatment was commenced.—W. W. TOWNSEND, *in Country Practitioner.*

**Puerperal Eclampsia.**—In one case of primipara, seventh month of utero gestation, I was called about six a.m. and found the patient in convulsions, insensible, unable to swallow, face almost wiry, pulse wiry, snapping, and so rapid that it was almost impossible to count it; os undilated and firm—convulsions recurring every twenty minutes, and lasting from one to three minutes. I had no anesthetic within reach, but did the only thing at hand—I bled her sixteen ounces by a full, free incision; it had no effect on the convulsions and did not change the pulse.

The indications were plainly to reduce the heart's action by some means, but how? She could not swallow; bleeding failed. I luckily thought of my inseparable companions, tr. verat. viride and the hypodermic syringe. I immediately injected three drops of the tincture under the skin, and waited for the effects to develop. At the expiration of the succeeding half hour, I was gratified to find the pulse reduced to one hundred and twenty; I then repeated the injection of three drops, and in twenty minutes the pulse was sixty, no convulsion had occurred since the first injection, great beads of perspiration stood upon the face, uterine contractions set in, and at the expiration of one hour, the patient was delivered of a male child, which, from the condition of the cuticle, had evidently been dead several days. Five times since the occurrence of the above case, I have had occasion to use the above treatment without bleeding, and with like favorable results. In late years cases have been reported where enormous doses of veratrum have been injected, but such action is useless, ridiculous and criminal. Better results are to be obtained by smaller doses.

That veratrum in its effects fills all the indications in sthenic cases of puerperal eclampsia is evident to any physician who understands its use. It is a fact generally admitted that quinine, given in small doses frequently repeated, has in most cases a better effect on the system than in large doses. After years of experience with veratrum, I am satisfied that small doses of the tincture, given at short intervals, either internally or hypodermically, has a better effect on the heart's action than when the dose is greater.—*Country Practitioner.*

**A Pleasant Remedy for Toothache.**—Our cook presented herself to me with a swollen cheek, asking for something to relieve the toothache, from which she had been suffering all night, and for which she refused to have the tooth extracted. As there was nothing of the usual kind at hand, I was on the point of telling her to call later at my office, or go to a dentist, when it occurred to my memory that there was in the house a vial of compound tincture of benzoin, which I had been using upon a young mother as a protection against sore nipples.

After cleansing the decayed tooth, I saturated a pledget of cotton lint with the tincture, and packed it well into the cavity, hoping this would suffice for the time; and bidding her come back in two or three hours if she was not relieved. I was turning away when she said it might not be necessary, perhaps, as the pain was already gone. Supposing her faith had a large share in the relief, I would not allow myself to think that the medicine had anything to do with the cure any more than so much hot water would have done.

But when I arrived at my office, two other patients were awaiting me with the same affliction, and I determined, by way of experiment, to use the same remedy. To my agreeable surprise, both patients declared themselves immediately relieved, and begged a vial of the tincture for future use.

During the winter a number of similar cases applied, and were instantly relieved by the same treatment, all expressing much satisfaction with the remedy.

In December I told my druggist of the discovery, and recommended him to sell it to any person applying for "toothache drops." This, he reports, he has done, and that every one seems delighted with the medicine.

Now, as this is the day of "small things," I trust that the "Brief" will pardon the size of this item, and duly credit me with the conviction of having contributed something useful, even if it be thought not at all brilliant.—T. C. OSBORN, M.D., *in Medical Brief.*

**Linseed and Oil in Skin Diseases.**—Dr. Sherwell claims great success in the employment of linseed and oil. He says:

1. If the patient were a male and had sound teeth, the seed itself was the best form in which to take it. The man could carry about ten ounces of this in his pockets, and would probably consume a teacupful in the course of a day. The ordinary domestic linseed was small and dark in color, and contained only about twenty per cent. of oil; while that from Bombay or Calcutta (which was the kind recommended), was larger, lighter in color, and contained about thirty per cent. of oil.

2. In the case of women or children, the ground seed, mixed with milk in the form of a porridge, was more desirable, and was unpalatable to very few persons.

3. In certain cases it could be given in the form of bread, although he did not consider this method quite so efficient as the others. The bread could be made by mixing linseed meal with flour in any proportion desired. This had been suggested to him by Dr. Piffard. (A loaf containing sixty per cent. of the meal was here presented to the Association, and was tasted by one or two of the members.)

When linseed was eaten, a natural emulsification was performed with the recent oil found in the stomach, and it had been established by chemists that a recent oil was much more active than one which had been long exposed to oxidation. The hulls also served to stimulate the peristaltic action of the intestines. He believed it had specific virtues in dry and scaly diseases of the skin, such as pityriasis rubra, ichthyosis, and dry eczema, both on account of its special action upon the sebaceous secretion and its effect in improving the general condition of the patient. Dr. Sherwell then gave in detail four cases of great obstinacy and severity, in which its curative influence was most happily shown. Two of them were cases of pityriasis rubra, one of pemphigus foliasis, and one of pemphigus vulgaris. He had also employed it with most marked benefit in four cases of ichthyosis, and had cured a large number of cases of chronic eczema with it. The seed was given internally in one of the forms above mentioned, and the oil applied externally. The lubricating effect of the latter was most admirable, and it had the advantage over most other oils of not becoming rancid when exposed to degraded epithelium. In eczema he was in the habit of wrapping the parts affected in a number of folds of linen saturated with it. He believed that flaxseed was a specific remedy for the sebaceous glands, increasing their secretion when it was diminished, and restoring it to its natural character when it had been altered by disease.—*N. Y. Med. Jour.*

**Inverted Toe Nail.**—The first thing to be done is, with a piece of glass, to scrape the top of the nail, along its middle, as thin as can possibly be borne. Then, with a sharp knife, cut from the middle of the free edge a v-shaped piece, carrying the apex of the v back as far as possible. The inverted corner is not to be disturbed, but is to be allowed to grow out squarely. For I have long regarded the cutting away of the corner of the nail as the most frequent exciting cause of the very difficulty in question. I say exciting cause, for I firmly believe, as Professor Gross intimates, that the main predisposing cause is heredity. I personally know a family in which the mother and three children have been thus afflicted. And in another family, closely related to the former, the mother and two children have been victims to the same annoyance. In these cases the children suffered in this way very early in life, thus lending considerable weight to the influence of heredity.

To return to the line of treatment, however, under the corner of the nail, which was to be allowed to grow out squarely, there must be placed a pledget of lint, of as large size as possible. This piece of lint must be thoroughly saturated with a mixture consisting of three grains of carbolic acid to one ounce of glycerin. Pains must be taken to have the lint wedged as tightly under the nail as possible. The lint must be renewed at least once daily. By these means I have been able to cure every case which has come under my charge. Some of these cases had for years, I was told, presented an ulcerating surface, covered with unhealthy granulation, and emitting an intensely fetid discharge.—DR. J. W. HICKMAN, *in Med. and Surg. Reporter.*

**Salivation of Pregnancy.**—Dr. P. C. Williams, in Baltimore Academy of Medicine, stated that one of his lady patients had exhibited this symptom to a most annoying degree. She seemed to have excessive salivation from the day of conception, and in spite of all treatment, including belladonna, it continued until she was relieved by labor. In this case, he spoke within bounds when he referred to quarts of saliva discharged in the twenty-four hours. This secretion continued when the pupils were dilated, and the throat felt parched from the physiological action of belladonna. For three successive pregnancies, this annoying symptom had shown itself. In one of the pregnancies, the fœtus died at the fourth month, but was carried to term, when a four months' child was born without decomposition, but bleached as if it had been kept in alcohol. The insalivation stopped with the death of the child, and did not return, notwithstanding the presence of the fœtus in utero.

Dr. B. B. Browne mentioned that, having failed in securing relief for one of his patients from belladonna, he had succeeded in checking the profuse salivary secretion by means of viburnum prunifolium. He considered its action to be a uterine sedative, allaying reflex irritation, and in that way stopping the inordinate salivary secretion.

**Pyrogallic Acid in Internal Hemorrhage.**—Dr. Vesey has been led to try the effects of pyrogallic acid in the treatment of internal hemorrhages from noticing its astringent effects when applied locally to the hands. He finds that it is of the greatest use when administered in grain doses in cases of hemoptysis, and suggests that it might be used in the form of spray. A combination of the remedy with ergot affords a very powerful means of arresting internal hemoırhage. Pyrogallic acid appears to have the following advantages: The dose is small; it does not disarrange the stomach in the way that the usual tannic or gallic acid mixtures do; it does not cause vomiting as iron and ergot mixtures; it is easily taken, and has no disagreeable after taste. It appears to be more rapid and certain than any of the remedies mentioned above, and far surpasses the time-honored acid infusion of roses or pil. plumbi cum opio. It dissolves readily in water or in spirit. A spirit solution of definite strength affords a convenient and ready method of administration. The grain doses are given every half hour till the hemorrhage is arrested, and may then, as a precautionary measure, be ordered every four hours, as long as the expectoration is tinged.—*Dublin Jour. of Med. Sci.*

**Antidote to Poison.**—If a person swallows any poison whatever, or has fallen into convulsions from having overloaded the stomach, an instantaneous remedy, most efficient and applicable in a large number of cases, is a heaping teaspoonful of common salt, and as much ground mustard, stirred rapidly in a teacupful of water, warm or cold, and swallowed instantly. It is scarcely down before it begins to come up, bringing with it the remaining contents of the stomach; and lest there be any remnant of the poison, however small, let the white of an egg or a teaspoonful of strong coffee be swallowed as soon as the stomach is quiet, because these very common articles nullify a larger amount of virulent poisons than any medicines.—*Brief.*

**Poisoning by Glanders.**—Glanders is a disease which the laws of every State should require to be stamped out by the instant destruction of the animal which has it. Quite a number of fatal cases in the human subject have been reported this year, both in England and America. It will not add to the comfort of the thousands of men who make daily use of trotting wagons and buggies to know that cases are related in which passengers in such vehicles have been infected with glanders by matter thrown off from the horses behind which they were seated.—*Med. and Surg. Reporter.*

**Arsenic as a Blood and Cardiac Tonic.**—Dr. Lockie, of the Cumberland Infirmary, calls attention to the remarkable effects obtained from the administration of arsenic in certain forms of anemia, in which iron and good food had failed to produce any benefit. Certain cases which have occurred in Dr. Lockie's practice, and of which the details are given, are cited to show that in cases of anemia approaching in gravity the so-called essential or pernicious anemia, the administration of liquor arsenicalis in five-minim doses was most advantageous. The author also supposes that the remedy might be useful in chorea and phthisis, since both are diseases associated with an anemic condition. In regard to arsenic as a cardiac stimulant, it is believed to be a valuable adjunct to digitalis, and in ordinary valvular disease of the heart, where there is a failure of compensation with its consequent results. Further, it seems to be of great value in fatty degeneration, and this in spite of the fact that recent experiments tend to show that fatty degeneration of the heart is one of the results of feeding animals with arsenic, *i. e.*, of the administration of arsenic in *large* doses.—*British Medical Journal.*

**Strangury from Blisters Prevented.**—Mr. G. Dannecy, chief pharmacien of the Bordeaux's hospitals, proposes in the *Bulletin de Therapeutique* the following improvement in the mode of applying blisters :

The following is the *modus operandi:* The blistering plaster, having been spread of the shape and size indicated, is dusted with a mixture of equal parts of coarsely powdered cantharides and carbonate of soda. The powder is then strongly pressed with the palm of the hand, to insure the adherence of the powder, and the surface is covered with oiled tissue paper. This method has been used exclusively for several years past, and only one complaint has ever been noted, no matter what may have been the size of the blisters. Besides, it has been observed that the vesicating effect was produced more rapidly than with the ordinary plaster.—*Drug. Circ. and Chem. Gazette.*

**Treatment of Hemorrhoids.**—Dr. W. M. Bemus, in Medical Brief, says: Seeing the appeal of Dr. G. M. Herndon for a remedy in hemorrhoids, where the ligature or knife be not deemed advisable, I should recommend a remedy that I have sometimes used with marked success. I should be pleased if the gentlemen would give it a trial, and report through the columns of this journal:

℞. Iodoform.............................30 grains.

Ext. hyoscyamus solid..................18 grains.

Cocoa butter, } q. s. to ft. suppositories.

Spermaceti, }

No. 10. Sig. Introduce one in the rectum night and morning.

This suppository, with the addition of belladonna solid extract in the proportion of one-half grain to a suppository, is a very satisfactory mode of treatment for enlarged prostate.

**Bryonia and Drosera for Hooping-cough.**—During the catarrhal stage, give the tincture of bryonia. During the spasmodic stage, give tincture of drosera. Under the action of the latter, the disease very rapidly subsides. So says M. Louve La-Mere in L'Annee Medicale.—*Journal of Materia Medica.*

**Resection of the Infra-orbital Nerve.**—Dr. Lasalle (*These de Paris*, and *Bulletin General de Therapeutique*) is of opinion that resection of the nerve is the only efficient way of healing, or at least soothing, inveterate chronic neuralgia. It ought to be performed either beyond the painful branches, or between them and the root of the nerve, but as far as possible from its termination. In cases of peripheric neuralgia the pain ceases almost instantaneously after the operation, but if its cause be in the nervous centres, the pain is only temporarily calmed. Having duly considered all these points, the author gives it as his opinion that the most favorable spot for the resection of the nerve is the orbital cavity, the operation being neither difficult nor the consequences dangerous. This situation is, therefore, preferable to the pterigo-maxillary fossa, where it is very difficult to perform the operation. It is also attended by much danger, so that it ought only to be made when the neuralgic pains have been caused by some traumatic affection of Meckel's ganglion. Dr Lasalle thinks it also advisable to remove the whole of the superior maxillary nerve instead of simply dividing or resecting it within the orbital cavity, as these operations only afford a momentary relief.—*London Med. Record.*

**Cheap Disinfectant.**—Dr. John Day, of Gelong, Australia, recommends for civil and military hospitals, and for the destruction of the germs of contagious diseases, a disinfectant composed of one part of rectified spirits of turpentine and seven parts of benzine, to each ounce of which five drops of essence of verbena have been added. The purifying and disinfecting properties are due to the presumed power of each of these ingredients to absorb oxygen from the atmosphere, and to convert it either into peroxide of hydrogen or ozone.

Clothing, furniture, papers, books, etc., can be saturated with the mixture without suffering detriment, and its action persists for an almost indefinite time, whether the disinfectant has been applied to a porous surface or not This may be proved by pouring upon any disinfected object a few drops of a solution of potassium iodide; the peroxide of hydrogen, which is being continually produced, frees the iodine and gives rise to dark brown spots.

The disinfectant may be applied with a sponge, or a brush; where it is possible, the object should be immersed in the liquid and then allowed to dry.—*Lyon Medical.*

**Suberine in Excoriated Nipples.**—The treatment advised by Dr. Brochard for sore and excoriated nipples is so simple that it deserves publicity.

As soon as an excoriation or a crack, no matter how small, appears upon the breast of a nursing woman, the nipple and areola should be washed with *pure water*, and after drying, powdered with *suberine*, or impalpable powder of cork. Suberine, which I always use for infants, is far preferable to lycopodium, which is an inert powder, because it contains tannin and is exceedingly cheap, an important consideration with many mothers. After applying the powder, the nipple is covered with a piece of gold-beater's skin placed over the nipple, thus allowing the baby to suck without causing pain to the nurse. After the infant has finished its meal, the nipple is again washed, powdered and covered.—*Detroit Lancet.*

**Vaccination as a Preventive of other Diseases than Variola.**—Among numerous interesting points in a report of a committee on vaccination and re-vaccination to the Medical Society of the county of King, New York, we find the statement that vaccination seems to exclude other diseases. In Wurtemberg, during five years, there were 325,646 births, of which number 208,322 were vaccinated. It has been assumed that three weeks is the average period during which the system is under the influence of the vaccine element. Of the number vaccinated, there were but 70 deaths during those three weeks, that is, one death in 3,000, while 35 would be the average number of deaths during the same number of weeks in children under one year. During the first three years the deaths would be 1,000, or 20 for the three weeks instead of *one*. Then, in children under one year, the death-rate, during the three weeks, is 35 times less than if the child were not under the influence of the vaccine, and under *three* years of age the average is *twenty* times less under similar circumstances.—*Cincin. Lancet and Clinic.*

**Therapeutic Effects of Bryony.**—Dr. Louvet-Lamare has published, in the *Annee Medicale de Caen*, some interesting observations on the effect of bryony and drosera in whooping-cough. In the first stage of the disease he administers tincture of bryony, in daily doses of fifteen minims, to children aged seven years; and states that it very quickly diminishes the bronchitis, stimulates the appetite, and does not create nausea. This plant seems to possess astringent properties, as is shown in the remark made by Barbier in his *Materia Medica*, vol. 3d, where he says that the peasant women are in the habit of taking, during some days, enemata made with the roots of bryony, when they cease to nurse their babies, and wish to prevent the secretion of milk in the mammæ.

Drosera has proved very efficient when whooping-cough has reached the paroxysmal stage. It was also employed formerly against dropsy and disease of the lungs, and is said to have been used with apparent success in phthisis.—*Med. Science.*

**Phosphide of Zinc.**—Gros, in *La France Medicale*, extols this article, and advises its use in nervous affections, and especially in hysteria; giving at the same time a long list of neuroses in which it has been successfully used by physicians in America and England. He says that, though hysteria is an affection strange in its termination, so many cures have been reported that we should prefer this remedy to others because of its promptness of action, its facility of administration, and its innocuousness. It is stated that, contrary to expectation, it is innocuous, because if a toxic dose is given, vomiting invariably occurs, which prevents the poisonous action of the drug. The best form for administration is the granule.—*Med. News.*

**Salicylic Acid Enemata in Dysentery.**—Dr. Berthold employs an enemata consisting of 1 gramme of salicylic acid, 300 grammes of distilled water, and alcohol q. s. In dysentery with tenesmus and bloody stools, he administers it every four hours. The tenesmus diminishes, and the number of stools is rapidly reduced, the fecal matters gradually acquiring their normal appearance, the temperature diminishing, and the appetite returning.—*Med. and Surg. Rep.*

**Arrowroot for Infants.**—Dr. Davis (Virginia Medical Monthly) says that there is, perhaps, no error more common than that of administering to the infant arrowroot, corn starch, tapioca, rice, oatmeal, sago, bread, crackers, or other starch foods, with the idea of thereby enriching the aliment provided by the substitution of cow's milk. This error is a very grave one, and the administration of this starch food is very injurious to infants, as it is not until after dentition that diastase is secreted by the salivary glands, and starch food remains in the stomach and intestines, not as food, but as a substance non-assimilable, foreign, and only disposed to irritate the delicate membranes. Dr. Routh, author of "Infant Feeding and its Influence on Life," says: "I cannot conceive anything more injurious than arrowroot feeding. I believe it is a cause of death of many infants."

**Iodide of Potassium in Vomiting.**—Dr. Formica Corsi states that he has cured with this drug cases of persistent vomiting that proved rebellious to the usual methods of treatment, and that he has known it to be equally successful in the hands of other practitioners. He cites the case of a woman who was suffering from typhoid fever and was at the same time in the second month of pregnancy. The vomiting resisted all the known anti-emetics. Finally he ordered a teaspoonful every hour and a half of a mixture consisting of half a grain of iodide of potassium in three ounces of water. On the following day the vomiting had ceased.

De Gine confirms this statement concerning the anti-emetic properties of the drug, and states further that, when given in doses of one-sixth to five-sixths of a grain per diem, it possesses decided laxative qualities.—*Gazette Obstetricale.*

**The Opium Habit.**—A correspondent of the New York Times, who professes to have been cured of the opium habit at the New York Inebriate Asylum, says that no drug can take the place of opium, and that those persons who profess to cure the opium habit by giving a substitute are mostly charlatans. He was not entirely deprived of the accustomed stimulant at once, but was allowed a small portion for two weeks in daily decreasing doses. After the opium had been abandoned, he was given belladonna by day and hydrate of chloral at night. The chloral was to induce sleep; this was reduced from what would be equivalent to about thirty grains of the salt, until at the end of the month it was nothing at all. By this time he could sleep without it. He was cured in a month, and, in the two months he remained there afterwards, gained in weight twenty pounds.—*Jour. Chem.*

**Treatment of Asthma.**—Dr. S. G. Armor says: In the treatment of asthma, the iodide of potassium is *the* remedy. But there is a class, in which there is an inflammatory element, that not unfrequently resists that treatment. For these he is in the habit of prescribing a preliminary treatment of divided doses of the bi-chloride of mercury, one-sixteenth to one-twentieth of a grain, for two or three weeks.

Under this drug the exudation becomes less viscid and tenacious, and the subsequent exhibition of the iodide of potassium becomes more efficient. Dr. Armor gave an account of two very stubborn cases, the cure of which was brought about under this plan.—*Med. News.*

**Successful Cataract Extraction in an Unpromising Case.** Dr. Chisolm reported a successful cataract extraction in a lady patient weighing nearly four hundred pounds. On account of her excessive plethora, the anterior chamber became filled with blood, making it very difficult to cleanse it before the lens was removed. For fear of inflammation, the patient was kept under the full effects of opium for two weeks. She had an uninterrupted convalescence, and did not suffer a moment's pain after the day of operation. At the expiration of fourteen days, she could read ordinary print, in evidence that a perfect success had followed upon the cataract extraction in so unpromising a patient.—*Va. Med. Monthly.*

**Cure of the Opium Habit.**—Dr. Osgood, of the Missionary Hospital at Foochow, has treated successfully several hundred cases of the opium habit by the following plan:

1. The total and absolute discontinuance of the opium from the beginning of treatment.
2. A trusty attendant to be with the patient day and night for the first three days.
3. Chloral hydrate for the first three nights, if required.
4. Good food, milk, raw eggs, brandy (in some cases), and chicken broth. (The above is taken in small quantities and frequently.)
5. In diarrhea, give two-drachm doses of a mixture of equal parts of tincture of catechu and tincture of ginger.—*Med. and Surg. Rep.*

**Bromide of Ammonium in too Frequent Menstruation.** J. R. Black, M.D., of Ohio, speaks in exalted terms, in the Ohio Medical Recorder, of the effectiveness of this treatment. Its administration should begin at least a week before the expected molimen, in doses of ten grains before each meal and at bed-time. Syrup of orange peel is a suitable vehicle for its administration. As a precautionary measure, the medicine should be taken before two or three subsequent and consecutive periods to prevent the possible reappearance of the abnormal issue. Iron and quinine we find to be our surest treatment for this menstrual disorder.—*Med. News.*

**Cancer of the Womb Treated with Bromine Injections.** (By DR. WILLIAMS, of Vienna.)—A woman, fifty years of age, had been treated for cancer of her womb, and the os had been removed. This was followed by new epithelial ulcers, which were touched with red-hot iron. After this, he used injections of bromine, one part to three of rectified alcohol, five to ten drops at a time. After three injections, the disease yielded. It is necessary to surround the parts with cotton or wool, saturated in an alkali, to escape injury from the bromine.—*Va. Med. Monthly.*

**Acute Glaucoma in a Woman 49 Years Old.**—She was treated by injections of corrosive sublimate in the nucha, and by frictions with glycerin, 100 parts, and iodine, one part. The injections were continued daily for two weeks, and then every other day for three weeks, when they, as well as the frictions, were discontinued. This inoffensive treatment cured the disturbance of the vitreous body and restored sight.—*Va. Med. Monthly.*

